# Consumer Trust Deficit and Repair Mechanisms in Food Safety Certification: Evidence from China’s Online Fruit Market

**DOI:** 10.3390/foods15213923

**Published:** 2026-11-02

**Authors:** Xiufan Ding, Jiping Sheng, Martina Sartoni, Alessandra Guidi

**Affiliations:** 1 School of Agricultural Economics and Rural Development, Renmin University of China, Beijing 100872, China; dingxiufan@ruc.edu.cn; 2 Department of Veterinary Sciences, University of Pisa, Viale delle Piagge 2, 56124 Pisa, Italy; martina.sartoni@vet.unipi.it (M.S.);

**Keywords:** food safety certification, commitment certificate of compliance (CCC), decoupling perception, institutional efficacy cognition, trust repair

## Abstract

This study examines why consumer trust in China’s food safety certification system remains deficient despite the widespread use of certification, and how this deficit can be repaired, with a focus on the Commitment Certificate of Compliance (CCC). Drawing on signaling theory, institutional trust theory, and decoupling theory, we develop a framework of signal failure under institutional trust deficit and test the asymmetric effects of decoupling perception and institutional efficacy cognition on consumer trust, willingness to buy (WTB), and willingness to pay a premium (WTP). Data were obtained from an online survey of 1047 consumers in 30 provincial-level regions of China through Wenjuanxing in 2026, with IP restrictions and attention checks to ensure data quality. We employ OLS, Probit, and ordered Probit models, together with Bootstrap mediation, propensity score matching, and moderation analyses. The results show that decoupling perception is negatively associated with trust (β = −0.0984, *p* < 0.01), WTB, and WTP, and this association remains robust after matching (ATET = −0.1027, *p* < 0.01). Institutional efficacy cognition is positively associated with trust (β = 0.0638, *p* < 0.01) and WTB, but not WTP. Perceived risk reduction, quality expectation improvement (indirect effect = 0.0371), and trust repair partially mediate these relationships. The repair effect is stronger on low-trust platforms and among consumers with high food safety awareness. Trust repair therefore requires a shift from issuing certificates to building institutional trust.

## 1. Introduction

High-quality development of agricultural products is a core task in China’s agricultural supply-side structural reform and rural industry revitalization. The 2024 No. 1 Central Document of China called for quality improvement and standardized production [1]. In 2024, national online retail sales of agricultural products exceeded 644.9 billion CNY, an increase of 15.8% [2]; online retail sales of physical goods reached 12,787.8 billion CNY, up by 6.5% and accounting for 26.5% of total retail sales of consumer goods, with food products growing by 16.0% [3,4]. By December 2025, the number of online shopping users reached 937 million, with a penetration rate of 83.2%, and monthly active users of instant retail applications stood at approximately 565 million [5]. In 2025, the national 12315 platform received 15.067 million complaints and reports about online shopping, accounting for 56.9% of the total, of which 2.385 million involved food safety issues, accounting for 11.7% [6]. Fresh agricultural product e-commerce is becoming an important engine for advancing high-quality agricultural development, but quality information asymmetry leads to the failure of high quality to command a high price and suppresses incentives for quality improvement. Pesticide residues, origin, and freshness of online fresh fruit have credence goods characteristics [7,8]. These attributes are difficult for consumers to identify before purchase and to verify after consumption, which can give rise to a market for lemons and constrain the high-quality development of agricultural e-commerce [9].

To address this, the Chinese government is building a quality signaling system centered on the Commitment Certificate of Compliance (CCC). The 2022 revision of the Agricultural Product Quality and Safety Law of the People’s Republic of China established traceability and commitment requirements [10]. The Administrative Measures for the Commitment Certificate of Compliance for Agricultural Product Quality and Safety, issued in 2025 and effective in 2026, define the CCC as a statutory written self-commitment by producers that their products meet safety standards, committing that no prohibited pesticides or veterinary drugs are used and that residues do not exceed the permitted limits, and require products within the stipulated scope to be marketed with the certificate [11]. The CCC differs from third-party certification, which relies on ex ante independent auditing, and from government-endorsed certification. It is a self-commitment constrained by ex post accountability and market access. If producers make false commitments, they face high fines, reputational losses, and even loss of market access, which enables the CCC to convey credible signals to consumers and makes it the most widely covering baseline compliance threshold in China’s food safety certification system.

Over the past decade, China has developed a multi-tiered framework centered on pollution-free, green, organic, and geographical indication products, known as the “Three Products and One Indication” [12]. However, consumer trust in the food safety certification system has not increased accordingly. Consumers generally doubt the authenticity of certification labels, and their trust in animal product certification is even lower [13]. Existing findings are inconsistent. More educated consumers are less influenced by labels, suggesting that institutional trust is more critical than labels themselves [14,15,16]. In the organic certification crisis, a special inspection in 2025 identified certification violations [17], reflecting a decoupling between formal certification and actual practice [18,19]. Green food faces the problem of certificates being purchased without substantive quality assurance [20]. Research on the aquatic food market also shows that consumers require a certification system covering the entire process [21].

However, the above studies mostly focus on third-party or government certification and rarely distinguish signal exposure from consumers’ cognition of institutional efficacy, leaving unexplained why a new certification signal may still fail even when it is widely displayed. To address this, this paper integrates three theories. Decoupling theory suggests that the current food safety certification system is gradually becoming a symbolic ritual serving legitimacy, and that consumers who perceive a disconnect between formal certification and actual practice develop defensive responses [18], which we term decoupling perception. Institutional trust theory holds that consumer trust in complex systems rests on beliefs about the supporting institutions and their enforcement capacity [22], and that institutional failure weakens the credibility of all signals. Signaling theory indicates that only when consumers recognize institutional constraints and penalties, and thus form institutional efficacy cognition that reconnects the symbol with the substance of certification, can the certification signal convey quality information again [23,24]. In addition, government governance faces incentive incompatibility [25], and information-disadvantaged consumers develop defensive skepticism after repeated deception, which amplifies the trust deficit [26]. Integrating these three theories, this paper develops a framework of signal failure under institutional trust deficit. This framework not only addresses the overreliance on a single theory in prior work but also directly yields the empirical core of this study, namely how decoupling perception leads to signal failure in the CCC context and whether and how institutional efficacy cognition can repair this trust deficit.

Accordingly, this paper proposes four progressive research questions. First, does decoupling perception reduce consumer trust, willingness to buy, and willingness to pay a premium. Second, can institutional efficacy cognition repair trust and enhance willingness to buy, and can this effect translate into willingness to pay a premium. Third, through which psychological pathways does institutional efficacy cognition operate. Fourth, under which platform and consumer characteristics is this trust-repair effect more pronounced. On this basis, this paper proposes the following hypotheses.

**H1a–H1c.**  *Decoupling perception negatively affects consumer trust (H1a), willingness to buy (H1b), and willingness to pay a premium (H1c).*

**H2a–H2c.**  *Institutional efficacy cognition positively affects consumer trust (H2a) and willingness to buy (H2b), but its effect on willingness to pay a premium is not significant (H2c).*

**H3.**  *Perceived risk reduction, quality expectation improvement, and trust repair mediate the relationship between institutional efficacy cognition and consumer trust, with quality expectation improvement showing the strongest mediating effect.*

**H4a–H4b.**  *The trust-repair effect of institutional efficacy cognition is stronger on low-trust platforms (H4a) and among consumers with high food safety awareness (H4b).*

## 2. Materials and Methods

### 2.1. Questionnaire Design and Research

This survey targeted consumers who purchase fresh fruit online in China. The survey was distributed nationwide via the Wenjuanxing platform from August 1 to 4, 2026. Using IP address restrictions, the sample covered 30 provincial-level administrative regions (excluding Tibet, Taiwan, Hong Kong, and Macau). The questionnaire was anonymous, and all information was used only for academic research. The completion time was approximately 8 min. A total of 1061 questionnaires were collected. After strict cleaning, 1047 valid samples were obtained. The cleaning process included: removing samples with no online fresh fruit purchase experience in the past year; performing logical checks on key variables; deleting missing values for marital status; and excluding three samples that selected “uncertain” for willingness to buy.

After consulting experts in the field of food science, two pre-investigations and adjustments, and discussion by the research group, the questionnaire was finally formed into six parts with a total of 41 questions. At the beginning of the questionnaire, the concept of online purchase of fresh fruits was defined, which refers to the fresh fruits (including domestic/imported, self-operated/third party, and live delivery) placed and delivered to the home through e-commerce platforms, online broadcast room, social media APP, cross-border e-commerce and other internet platform types, excluding processed products such as fruit cutting boxes. The specific structure of the questionnaire is as follows:

Part 1: Consumption preferences and behaviors (online purchase experience, frequency, spending, fruit types, platforms, key factors, ordering habits).

Part 2: Quality safety and problem experiences (overall evaluation, information sources, encountered problems, handling of substandard fruit).

Part 3: Trust and behavioral responses (trusted platform types vs. store trust, responses to quality discrepancies, most trusted entity).

Part 4: Consumer values (time preference, health awareness, food safety awareness, impact of after-sales commitments).

Part 5: Commitment Certificate of Compliance (policy background, definition, issuance, familiarity, perceived information, signal type, checking behavior, trust evaluation, influencing factors, views on self-commitment, choices, premium, reaction to poor quality).

Part 6: Background information (residence, area type, gender, age, marital status, family composition, education, income, occupation).

To ensure the alignment between measurement items and theoretical concepts, the core items were designed based on signaling theory, institutional trust theory, and decoupling theory. Content validity was established through a review by three experts in food science and agricultural economics. A pilot test with 26 consumers was then conducted to refine item wording and options. The internal consistency of the multi-item continuous scales (e.g., consumer trust) was evaluated using Cronbach’s α, yielding a coefficient of 0.674. The final questionnaire was administered anonymously and included attention check questions to ensure data quality. It covered multidimensional measurements of behavior, cognition, trust, and willingness to pay a premium, providing a solid foundation for empirically testing the framework of signal failure under institutional trust deficit.

### 2.2. Econometric Model

To empirically test the theoretical framework of signal failure under institutional trust deficit, we model consumer trust, willingness to buy, and willingness to pay a premium as functions of two key explanatory variables: decoupling perception and institutional efficacy cognition, together with a set of individual-level controls. The framework posits that decoupling perception represents symbol–substance decoupling, which triggers defensive processing and suppresses trust, whereas institutional efficacy cognition represents symbol–substance recoupling, which restores the signaling function and enhances trust [27]. We therefore expect asymmetric effects of these two variables across the three outcome variables. Furthermore, to ensure clarity and reproducibility, the core explanatory variable decoupling perception is constructed as a binary variable. It is coded as 1 if the consumer perceives a disconnect between the formal certification label and actual product quality (i.e., the symbol–substance decoupling), and 0 otherwise. The consumer trust index is constructed by calculating the mean score of its multiple items, whereas institutional efficacy cognition is measured by a single item.

To test the proposed hypotheses, we model consumer trust, willingness to buy, and willingness to pay a premium as functions of decoupling perception and institutional efficacy cognition, together with a set of control variables. Because the three dependent variables differ in nature, we employ OLS for the continuous trust index, Probit for the binary willingness to buy, and Ordered Probit for the ordinal willingness to pay a premium. In addition, we also use Logit and Ordered Logit for stability test. The baseline specifications are given by:

(1) $${Trust\_Index}_{i}=\alpha_{0}+\alpha_{1}{Decoupling\_Perception}_{i}+\alpha_{2}{Institutional\_Efficacy\_Cognition}_{i}+\gamma {Controls}_{i}+\varepsilon_{i}$$

(2) $$P\left({WTB}_{i}=1\right)=\Phi (\beta_{0}+\beta_{1}{Decoupling\_Perception}_{i}+\beta_{2}Institutional\_Efficacy\_Cognition+\theta {Controls}_{i}+\mu_{i})$$

(3) $${WTP}_{i}^{*}=\delta_{0}+\delta_{1}{Decoupling\_Perception}_{i}+\delta_{2}{Institutional\_Efficacy\_Cognition}_{i}+\varphi {Controls}_{i}+v_{i}$$

where ${WTB}_{i}\;$is an unobserved latent variable mapped into the observed ordinal ${WTB}_{i}$ through thresholds $\mu_{1},\;\mu_{2},\;\mu_{3},\;and\;\mu_{4}$, and Φ(⋅) is the standard normal cumulative distribution function. ${Controls}_{i}$ includes online purchase frequency (Freq), health awareness (Health), time preference (Time_Pref), age (Age), gender (Gender), and marital status (Marriage). Based on the theoretical framework, we expect $\alpha_{1},\beta_{1},\delta_{1}<0$ and $\alpha_{2},\beta_{2},\delta_{2}>0$, while the coefficient of institutional efficacy cognition in the WTP model may be insignificant because recoupling cognition can repair trust and willingness to buy but may not directly translate into a price premium [28].

To uncover the underlying “black box” mechanism of how institutional efficacy cognition translates into consumer trust, a mediation analysis is theoretically appropriate. To test this mechanism, we follow Baron and Kenny and estimate a set of recursive equations [29]:

(4) $$Y_{i}=cX_{i}+\gamma {Controls}_{i}+\varepsilon_{i}$$

(5) $$M_{i}=aX_{i}+\gamma {Controls}_{i}+\varepsilon_{i}$$

(6) $$Y_{i}=c^{\prime }X_{i}+bM_{i}+\gamma {Controls}_{i}+\varepsilon_{i}$$

where X is institutional efficacy cognition, M denotes the mediators including perceived risk reduction, quality expectation improvement, and trust repair, and Y is the consumer trust index. Because the traditional Sobel test assumes a normal distribution of the indirect effect, which is often violated in finite samples, we use the Bootstrap method with 5000 replications to empirically construct the confidence intervals. A mediation effect is established if the bootstrap confidence interval excludes zero. Based on cognitive dissonance theory, we expect that Institutional efficacy cognition reduces perceived risk, enhances quality expectations, and promotes trust repair, with quality expectation improvement being the strongest mediator [30].

For the moderation analysis, we estimate:

(7) $$Y_{i}=\lambda_{0}+\lambda_{1}{Institutional\_Efficacy\_Cognition}_{i}+\lambda_{2}Z_{i}+\lambda_{3}\left({Institutional\_Efficacy\_Cognition}_{i}\times Z_{i}\right)\;+\gamma {Controls}_{i}+\varepsilon_{i}$$

where Z includes platform type, education, income, and food safety awareness, with food safety awareness mean-centered. The coefficient $\lambda_{3}$ captures the moderating effect. We expect $\lambda_{3}<0$ for platform type because the trust-repair effect is stronger on low-trust platforms, and $\lambda_{3}>0$ for food safety awareness because consumers with higher food safety awareness are more efficient in converting cognition into trust [31]. Education and income are included as exploratory moderators.

Finally, in observational survey data, consumers’ decoupling perception is not randomly assigned, which may lead to self-selection bias. To address this endogeneity and construct a valid counterfactual framework, we employ propensity score matching (PSM). The propensity score is estimated via a Logit model:

(8) $$P\left(D_{i}=1|{Controls}_{i}\right)=\Lambda \left(\alpha \cdot {Controls}_{i}\right)$$

where $D_{i}={Decoupling\_Perception}_{i}$. We then calculate the average treatment effect on the treated (ATET):

(9) $$ATET=E\left[Y_{1i}-Y_{0i}|D_{i}=1,P\left(X_{i}\right)\right]$$

using 1:3 nearest-neighbor matching. Balancing tests are conducted to ensure that the standardized bias of all covariates falls below 10% after matching. When signal accuracy is costly, the platform has an incentive to actively erode signal quality, even at a cost, which erodes buyer trust and collapses trade [32]. If the negative effects of decoupling perception on trust, willingness to buy, and willingness to pay a premium remain significant after propensity score matching, the findings support the interpretation that signal failure under institutional trust deficit is not driven by sample selection.

## 3. Results

### 3.1. The Overall Situation of Chinese Consumers Buying Fresh Fruit Online

Survey results show that China has a large user base for online fresh fruit shopping. Regarding purchasing habits, 98.68% of respondents have purchased fresh fruit online in the past year, and 78.7% purchase at medium-to-high frequencies of 1 to 5 times per month. The average spending per order is concentrated in the 30–99 CNY range, accounting for 65.5% of the sample. The most frequently purchased categories are citrus fruits at 73.42%, tropical fruits such as bananas and mangoes at 62.02%, and apples and pears at 60.79%.

In terms of platform preferences, instant fresh e-commerce and traditional e-commerce are the primary channels with usage rates exceeding 77% each, followed by livestreaming e-commerce at over 50% and community group buying at approximately 32.8%. When choosing platforms, consumers place greater emphasis on price affordability at 67.58%, convenience at 56.83%, and freshness at 46.94%, while paying relatively less attention to brand or origin at 19.32%. Quality judgments rely primarily on merchant reputation at 61.92%, with limited reference to certification labels at 46.65%. Overall evaluations of online fruit quality are generally positive, with 62.02% rating it as good or very good. However, practical issues remain common, primarily unripe, deteriorated, or rotten fruit at 55.7%, inconsistency with descriptions at 49.67%, and unstable quality in repeat purchases at 40.72%.

### 3.2. Descriptive Statistics of the Sample

The valid sample for this survey consisted of 1047 consumers. As shown in Table 1, the sample was dominated by young and middle-aged adults: those aged 18–29 and 30–44 accounted for 30.85% and 60.46%, respectively, together comprising 91.31%, while the 45–59 and 60-and-above groups represented only 8.02% and 0.67%. In terms of gender, females made up 58.07%, males 41.64%, and 0.29% did not report a clear gender, indicating a relatively higher proportion of female consumers. Regarding marital status, married respondents accounted for 76.31% and unmarried respondents for 23.69%, with married individuals forming the majority. In terms of income, based on the classification in the table (monthly income below 12,000 CNY defined as low-income and above 12,000 CNY as high-income), the low-income group accounted for 75.74% and the high-income group for 24.26%, suggesting that the sample was mainly composed of low-income consumers. With respect to education, the low education level group represented 90.35% and the high education level group only 9.65%, indicating that the sample was predominantly composed of individuals with lower education levels. Overall, the sample is characterized by a structure dominated by young and middle-aged, female, married, and low-income consumers, and aligns with the general demographic profile of the current online fresh fruit consumer group.

The descriptive statistics of the key variables in Table 2 show that consumer trust and willingness to buy certified fruit are generally high, whereas the willingness to pay a premium remains only moderate, suggesting that trust does not fully translate into additional payment. Meanwhile, the level of decoupling perception is relatively elevated, implying that many consumers have encountered the certification label but lack a deeper understanding of its institutional underpinnings, thereby providing a basis for examining signal failure. Institutional efficacy cognition, in turn, is moderate, reflecting insufficient consumer understanding of institutional effectiveness and highlighting the need for trust repair. The three mediators all exhibit relatively high means, indicating positive psychological responses in terms of perceived risk reduction, quality expectation improvement, and trust repair. In addition, the moderators and control variables show sufficient variation for regression analysis. Overall, the sample is characterized by a structure dominated by young and middle-aged, female, married, and low-income consumers, which aligns with the general demographic profile of frequent online fresh fruit shoppers reported in industry data.

### 3.3. Baseline Regression Results

Table 3 presents the baseline regression results. The models use OLS for the consumer trust index (CTI), Probit and Logit for willingness to buy (WTB), and ordered Probit and ordered Logit for willingness to pay a premium (WTP). Because Probit and ordered Probit coefficients are scaled by the latent-variable variance and cannot be read directly as marginal effects, Table 4 additionally reports the average marginal effects (AMEs) of the core explanatory variables with their 95% confidence intervals; for the ordered Probit model, AMEs are computed for each WTP category.

Decoupling perception has a negative and significant effect in all five specifications. Its coefficient is −0.0984 for CTI (*p* < 0.01). For willingness to buy, it is −0.2157 in the Probit model and −0.3772 in the Logit model (*p* < 0.05),with an AME for WTB of −0.052 in the Probit model (95% CI [−0.0979, −0.0061]). For willingness to pay a premium, it is −0.2287 in the ordered Probit model and −0.3924 in the ordered Logit model (*p* < 0.01), with an AME for the highest WTP category (WTP = 5) of −0.0032 in the ordered Probit model (95% CI [−0.0061, −0.0003]). These results suggest that mere exposure to the certificate symbol, without understanding its institutional constraints, reduces consumer trust, willingness to buy, and premium willingness.

Institutional efficacy cognition shows a different pattern. It significantly increases CTI (0.0638, *p* < 0.01) and willingness to buy (0.1869 in Probit; 0.3351 in Logit; *p* < 0.01), with an AME for WTB of 0.0451 in the Probit model (95% CI [0.0259, 0.0642]). However, its effects on willingness to pay a premium are negative but insignificant (−0.0337 in ordered Probit; −0.0390 in ordered Logit), and the corresponding AMEs for the WTP categories are likewise insignificant, with 95% confidence intervals that include zero (Table 4). This asymmetric result indicates that institutional efficacy cognition can repair trust and encourage buying, but it cannot yet translate directly into a higher willingness to pay a premium.

Among the control variables, health awareness and online purchase frequency are positive and significant in all reported models. For example, health awareness has coefficients of 0.4310 for CTI, 0.5256 for willingness to buy in the Probit model, and 0.2154 for willingness to pay a premium in the ordered Probit model. Online purchase frequency has coefficients of 0.0801, 0.1674, and 0.3260 in the same models. Time preference is insignificant, and most other demographic controls are also insignificant. The models show acceptable fit: R^2^ is 0.2516 for CTI, pseudoR^2^ is around 0.09 for willingness to buy, and pseudoR^2^ is around 0.04–0.05 for willingness to pay a premium. All F or LR chi-square tests are significant at the 1% level. Overall, the AMEs in Table 4 are consistent in sign and significance with the baseline coefficients in Table 3, and the baseline results support the study’s framework of signal failure under institutional trust deficit.

### 3.4. Test for Endogeneity

In order to deal with the possible self-selection bias of decoupling perception, this paper adopts propensity score matching (PSM) for endogeneity processing. Table 5 reports the average treatment effects (ATET) estimated separately with the 1:3 nearest neighbor matching method for the three dependent variables.

Table 5 reports the robustness test results using Propensity Score Matching (PSM) to address potential self-selection bias in decoupling perception. The results show that decoupling perception has a significant negative average treatment effect on the treated (ATET) across all three dependent variables. Specifically, the ATET is −0.1027 (*p* < 0.01) for the consumer trust index, −0.0636 (*p* < 0.05) for willingness to buy, and −0.1045 (*p* < 0.1) for willingness to pay a premium, with 95% confidence intervals of [−0.1745, −0.0309], [−0.1272, 0.0001], and [−0.2228, 0.0139], respectively. It is worth noting that for willingness to buy, the upper bound of the 95% confidence interval slightly touches zero (0.0001); thus, this specific effect should be interpreted as marginally significant. Overall, these findings are broadly consistent with the baseline regression results, indicating that the negative effects of decoupling perception on consumer trust and premium willingness remain robust after controlling for observable selection bias, while the effect on willingness to buy is marginally robust.

**Figure 1:**
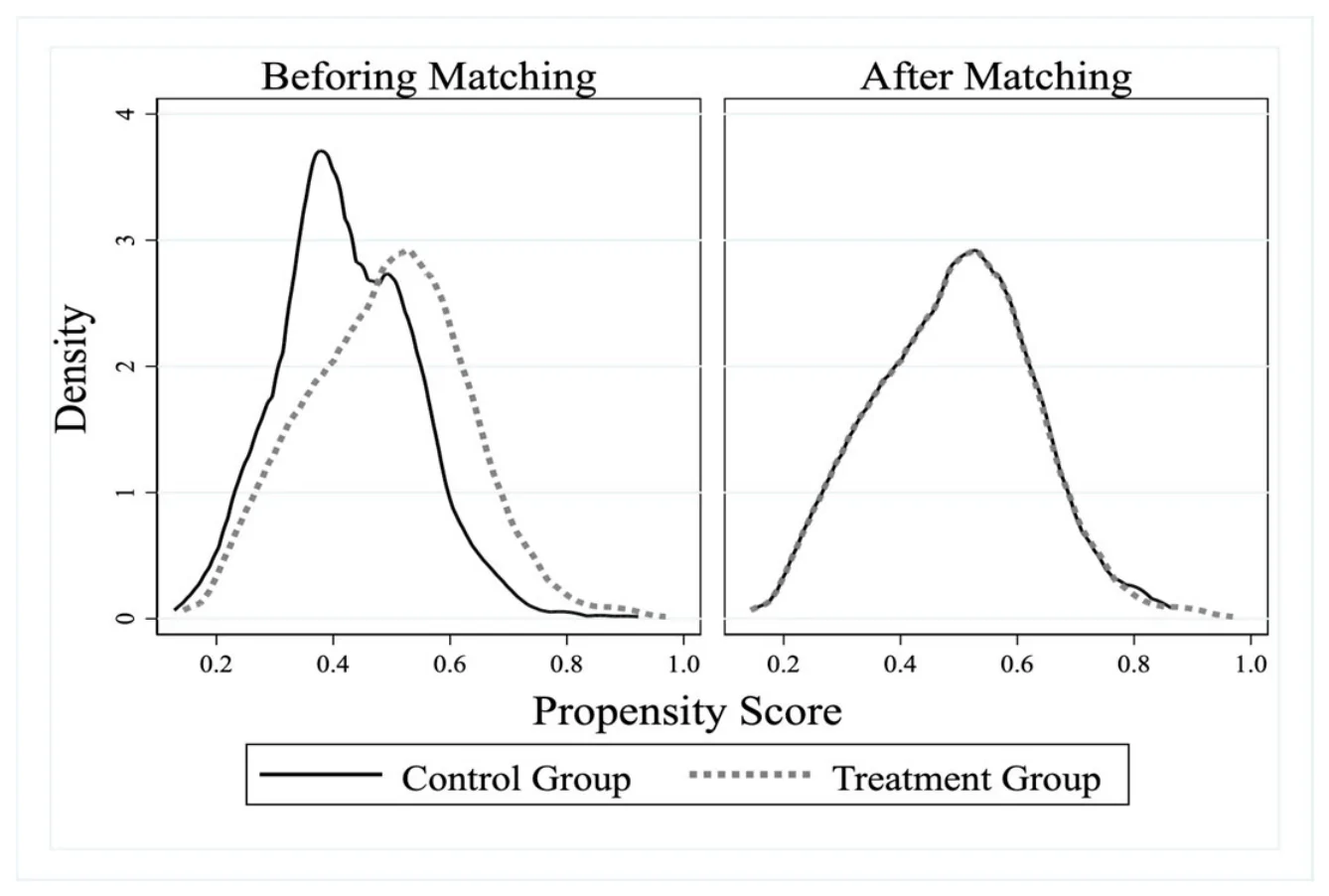
Kernel density plots before and after propensity score matching.

Table 6 presents the balance test results of covariates before and after propensity score matching. Prior to matching, significant differences existed between the treated and control groups, particularly in Online Purchase Frequency (standardized bias = −0.433) and Health Awareness (standardized bias = −0.321), indicating substantial self-selection bias. After 1:3 nearest-neighbor matching, the standardized biases for most covariates decreased substantially. Specifically, Online Purchase Frequency, Health Awareness, Time Preference, Gender Group 2 (Female), Age Group 2 (30–44), Age Group 3 (45–59), and Marital Status all achieved absolute standardized biases below 10%, with variance ratios close to 1. However, for Age Group 4 (60 and above), the standardized bias after matching is 10.2% (0.102), slightly exceeding the conventional 10% threshold, and its variance ratio is 3.567. This imperfection stems from the fact that there are only 7 respondents aged 60 and above in the entire sample. The extremely limited sample size of this specific subgroup reduces matching precision. Nevertheless, given that the vast majority of covariates achieved good balance, and the overall propensity score distributions become highly overlapping (as shown in Figure 1), the overall PSM balance test is considered satisfactory. PSM mitigates observable selection bias but does not establish causality, the results should be interpreted as robustness checks.

### 3.5. Mediating Effect Analysis

In order to explore the internal transmission mechanism of institutional efficacy cognition affecting consumer trust, this paper tests the mediating effect based on the three-step method of Baron and Kenny [30], and uses the Bootstrap method (5000 repeated samples) to verify the significance of the indirect effect. Table 7 reports the regression results with institutional efficacy cognition as the core independent variable and perceived risk reduction, quality expectation improvement and trust repair as mediating variables.

Table 7 reports the three-step mediation results. Institutional efficacy cognition has a significant positive total effect on consumer trust (c = 0.0653, *p* < 0.01). It also significantly reduces perceived risk (a1 = 0.0493, *p* < 0.05), improves quality expectation (a2 = 0.0795, *p* < 0.01), and promotes trust repair (a3 = 0.0772, *p* < 0.01). When each mediator is added separately, all three mediators show significant positive effects on trust: perceived risk reduction (b1 = 0.3760, *p* < 0.01), quality expectation improvement (b2 = 0.4667, *p* < 0.01), and trust repair (b3 = 0.4780, *p* < 0.01). Meanwhile, the direct effect of institutional efficacy cognition drops notably. This pattern indicates partial mediation. Table 8 further confirms these mediation effects using bootstrap with 5000 replications. The indirect effects are significant for perceived risk reduction (0.0185, 95% CI [0.0038, 0.0332]), quality expectation improvement (0.0371, 95% CI [0.0224, 0.0522]), and trust repair (0.0369, 95% CI [0.0190, 0.0540]). None of the confidence intervals contains zero. Among the three paths, quality expectation improvement has the largest indirect effect. This is consistent with the interpretation that rebuilding consumers’ confidence in the quality guaranteed by the certification system is an important channel through which institutional efficacy cognition is associated with trust. These indirect effects are statistical pathways identified by Bootstrap mediation; they are consistent with, but do not establish, causal mediation.

### 3.6. Analysis of Heterogeneity

The baseline regression examined the average effects of decoupling perception and institutional efficacy cognition on consumer trust, but the repair effect of the latter may vary across contexts and groups. According to the framework of signal failure under institutional trust deficit, the more severe the institutional trust deficit, the stronger the marginal repair effect of recoupling cognition. To identify these boundary conditions, we introduced platform type, food safety awareness, education, and income as moderators and included their interaction terms with institutional efficacy cognition in the baseline model.

Table 9 shows the moderating effects of platform type, education, income, and food safety awareness on the relationship between institutional efficacy cognition and the consumer trust index. The interaction between institutional efficacy cognition and food safety awareness is positive and significant at the 1% level (β = 0.0931). The interaction between institutional efficacy cognition and platform type is negative and significant at the 10% level (β = −0.0167), where platform type is coded as 1 for high-trust platforms and 0 for low-trust platforms. The interactions between institutional efficacy cognition and education (β = −0.0124) and income (β = −0.0022) are not statistically significant.

**Figure 2:**
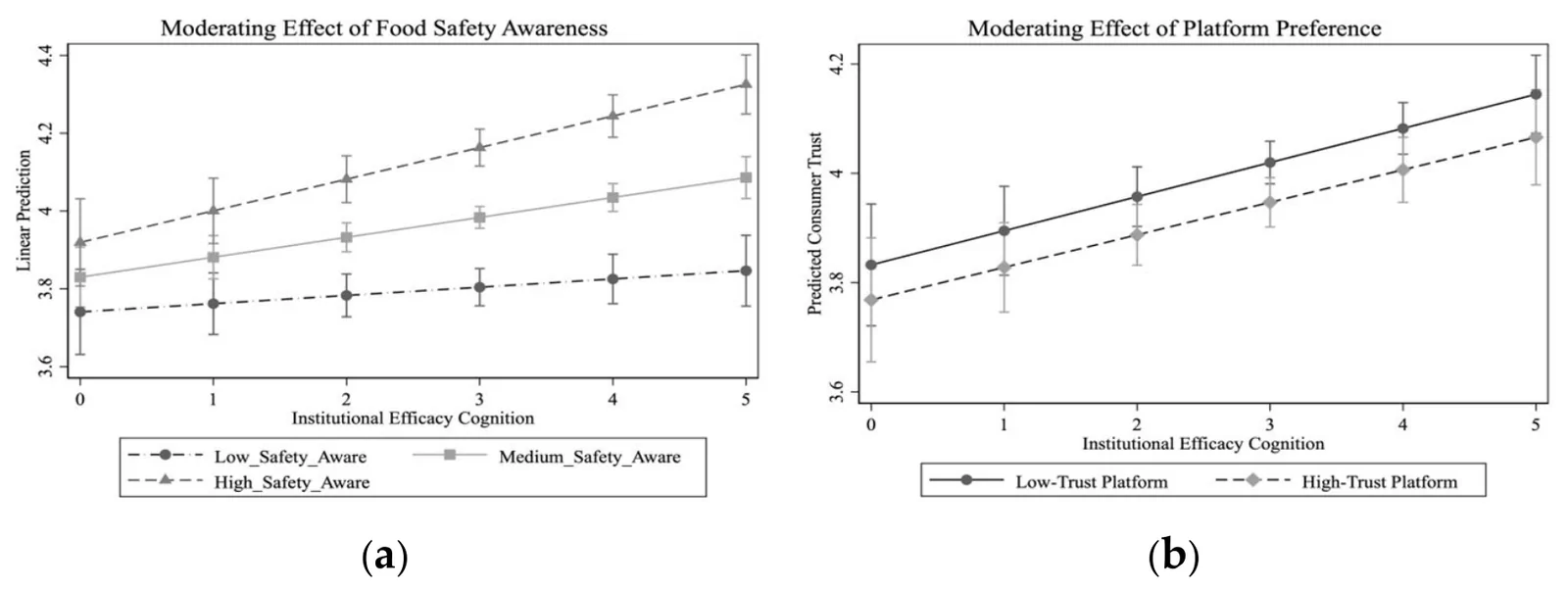
Slope plot of moderating effect: (**a**) Moderating effect of food safety awareness on the relationship between institutional efficacy cognition and consumer trust index; (**b**) moderating effect of platform type preference on the relationship between institutional efficacy cognition and consumer trust index.

As illustrated in Figure 2a, the slope for consumers with high food safety awareness is steeper than that for those with low awareness, indicating that the positive effect of institutional efficacy cognition on trust is stronger among consumers who are more sensitive to food safety information. This aligns with the theoretical expectation that consumers with higher food safety awareness are more efficient in converting institutional efficacy cognition into trust.

Regarding platform type, the interaction term is negative and significant at the 10% level (β = −0.0167). Since high-trust platforms are coded as 1 and low-trust platforms as 0, this negative coefficient indicates that the positive marginal effect of institutional efficacy cognition on trust is larger on low-trust platforms. This pattern is visually confirmed in Figure 2b, where the slope for low-trust platforms is notably steeper. This suggests that in environments where institutional trust is more severely lacking, such as livestreaming e-commerce and community group buying, the certificate functions as a scarce hard signal, thereby playing a stronger marginal role in trust repair.

Meanwhile, the interactions between institutional efficacy cognition and education and income are not statistically significant (*p* = 0.415 and *p* = 0.842, respectively). This indicates that the trust-repair effect depends more on individual psychological traits (e.g., food safety awareness) and external institutional environments (e.g., platform type) than on demographic characteristics. Overall, the heterogeneity analysis further delineates the boundary conditions of the signal failure under institutional trust deficit framework.

## 4. Discussion

### 4.1. Theoretical Explanation of Signal Failure Under Institutional Trust Deficit

This study finds that decoupling perception is negatively associated with consumer trust, willingness to buy, and willingness to pay a premium, whereas institutional efficacy cognition is positively associated with trust and willingness to buy, an asymmetry consistent with signal failure under an institutional trust deficit. From a signaling perspective, senders convey quality through costly signals, and receivers interpret them to narrow the information gap [23]; the theory does not assume costless interpretation, since interpretation costs and differences are central to it [24]. Interpretation demands time, expertise, and cognitive ability, which vary across receivers and rise in noisy markets. Food certification is such a signal: consumers tend to view enterprise-driven certifications as marketing strategies rather than quality commitments [33], so skeptical processing precedes institutional evaluation, a state captured by our decoupling perception variable.

These results align with evidence that certification-related trust is heterogeneous among urban Chinese consumers [13], food-safety perceptions shape willingness to pay for certified food [34], institutional trust affects repurchase intentions [14], and trust is institutionally produced [22]. The certification signal is thus embedded in consumers’ trust in the institutional environment, so the repair pathway we identify is a general mechanism. Nevertheless, our results differ from two strands of research. First, unlike studies treating certification awareness as uniformly beneficial, we find that mere exposure without institutional understanding is not merely ineffective but potentially harmful; a likely explanation is that signal interpretation depends on institutional trust [35]. Second, unlike classical signaling theory, where interpretation costs are endogenous, an institutional trust deficit raises them so sharply that a costly signal no longer conveys credibility, the same certificate being read as cheap and self-serving. We also extend producer-side decoupling [19] to consumer cognition, echoing the trust paradox [32], in which over-reliance on labels can reduce conversion when trust is damaged. These differences delimit when signal failure occurs, underscoring that rebuilding institutional credibility is the crux of repair.

The problem is not a shortage of information but an institutional trust deficit that undermines consumers’ ability and willingness to interpret certification signals. These conclusions concern trust and willingness to buy alone, and we make no claim about price premiums or actual purchase behavior. From a practical standpoint, repairing this problem requires strengthening the perceived institutional constraints behind certification through credible enforcement, transparency, and traceability.

### 4.2. The Essence of Cognitive Repair: From Decoupling to Recoupling

The positive association between institutional efficacy cognition and trust and willingness to buy corresponds to a process in which cognitive repair reconnects symbol and institutional substance: if consumers distrust the certifying institution, the signal appears cheap and decoupled from actual quality [36]. This is consistent with prior evidence that institutional trust and label knowledge significantly strengthen eco-label trust (0.351, 0.357) [37]; once consumers understand counterfeiting costs and government traceability, the label becomes visible evidence of institutional constraints [34]. Chinese consumers likewise regard certified products as safer and of higher quality, with trust determining their willingness to pay [38].

Mediation analysis identifies three pathways, namely perceived risk reduction (0.0185), quality expectation improvement (0.0371), and trust repair (0.0369), among which quality expectation improvement shows the largest indirect effect, corroborating the interpretation that confidence in system-guaranteed quality is an important channel through which institutional efficacy cognition is associated with trust, and extending prior work in this area [10]. Yet institutional efficacy cognition is not significantly associated with premium willingness, possibly because self-commitment signals are less verifiable than third-party certification [38]; government certification is likewise trusted more than private or self-declared schemes, which explains the credibility gap of the CCC [39], so premium realization calls for stronger institutional constraints and transparency [38].

Repair also depends on consumer characteristics. Higher food-safety knowledge strengthens trust in certified labeled food and official platforms, making knowledge a cognitive amplifier [40]; certification trust rests on perceived institutional legitimacy and historical performance and erodes when certifiers fail to uphold standards [41], while signal effectiveness likewise hinges on certifier credibility and the absence of conflicts of interest [33]. Overall, people’s perceptions of institutional effectiveness link to trust and behavior only when those perceptions rest on genuinely credible institutions; moreover, these findings apply solely to trust and willingness to buy.

### 4.3. Heterogeneity in Trust Repair

The trust-repair effect of institutional efficacy cognition is stronger on low-trust platforms and among consumers with high food safety awareness, whereas education and income show no significant moderation, a pattern consistent with the severity of the institutional trust deficit and recoupling efficiency. On low-trust platforms such as livestreaming e-commerce and community group buying, consumers lack alternative quality signals such as platform endorsement or accumulated merchant reputation, so the certificate becomes a scarce hard signal and is associated with a greater marginal repair effect. This aligns with evidence that certification signals matter more under weak regulation and high information asymmetry [42,43], and that platform certification increases seller sales in food e-commerce, particularly when reputation mechanisms are weak [44]. Within the signal failure framework, a more severe institutional trust deficit is associated with a higher marginal utility of recoupling cognition, one interpretation being that building high-quality agricultural product regional brands can serve as a credible institutional signal, helping consumers compensate for missing quality cues [45].

Consumers with high food safety awareness likewise show patterns consistent with higher repair efficiency; one interpretation is that they are more sensitive to the institutional trust deficit and more motivated to internalize institutional efficacy cognition. Cognitive dissonance theory suggests they experience stronger psychological imbalance when facing uncertified products or doubtful certificates, and thus actively seek and process institutional information, consistent with prior work on food safety awareness and information processing [46,47]. Higher food safety knowledge also increases awareness of certified labeled food and trust in official sources [48], and limited attention to food safety information makes the motivation to process it critical.

Education and income show insignificant moderating effects. Highly educated consumers may scrutinize self-commitment signals more rationally, while high-income consumers remain conservative despite greater payment capacity, indicating that institutional trust repair depends more on psychological traits such as food safety awareness and on external environments such as platform reputation than on cognitive or economic capacity [49]. Although income and education are associated with willingness to pay a premium for certified food, they operate independently of trust-repair mechanisms; institutional trust, rather than demographic characteristics, primarily drives willingness to buy for certified traceable food [14]. These results further define the boundary conditions of signal failure under an institutional trust deficit: repair depends not on the ability to understand, but on the motivation to pay attention and environmental support.

### 4.4. Limitations and Future Research

This study has several limitations that point to future research directions. First, the cross-sectional data limit causal inference; although propensity score matching addresses observable selection bias, omitted variable bias and reverse causality cannot be fully ruled out. Future research could employ panel data or quasi-experimental designs, or construct instrumental variables. Second, the measurement of institutional efficacy cognition relied on a single item, and the consumer trust scale yielded a Cronbach’s α of 0.674, slightly below the 0.70 threshold. This may stem from the exploratory nature of the scale and the limited pilot sample size. Future research should refine these scales to improve reliability. Third, the mediating chain from institutional efficacy cognition to trust repair requires experimental validation, such as information intervention experiments, to test the causal effects of recoupling. Additionally, as all data were collected from a single self-report survey, common method bias (CMB) may exist. This was mitigated by ensuring respondent anonymity and randomizing item order in the survey design.

## 5. Policy and Conclusions

### 5.1. Conclusions

The empirical evidence reveals a structural asymmetry in the formation and repair of consumer trust within certification systems. Superficial exposure to certification symbols can inadvertently activate decoupling perception and undermine consumer confidence. By contrast, genuine trust repair is fundamentally driven by institutional efficacy cognition, which represents consumers’ recognition of substantive institutional constraints. This transition from symbolic exposure to institutional recognition is not merely an informational process but a psychological shift from decoupling to recoupling. Through this shift, perceived risk is mitigated and quality expectations are restored. Consequently, bridging the trust deficit in food safety certification necessitates a paradigm shift in governance, moving beyond the conventional promotion of certificate issuance toward the cultivation of institutional credibility. Ultimately, the effectiveness of any quality signal is intrinsically bound to the perceived legitimacy of the enforcement mechanisms behind it.

This dependence on context also means that policy interventions must be precisely tailored to specific environments. The asymmetric efficacy of institutional cognition is more pronounced where institutional trust is most acutely lacking, such as on low-trust platforms, and among consumers with elevated food safety awareness. This suggests that trust building is a highly targeted endeavor rather than a uniform, one-size-fits-all approach. Restoring consumer trust requires strategically concentrating governance resources to amplify institutional credibility where it is most critically needed. These insights collectively provide a robust theoretical foundation for optimizing institutional design, integrating platform credit governance, and dynamically monitoring the translation of formal certification into substantive consumer trust.

### 5.2. Policy

Our findings indicate that restoring the effectiveness of certification signals depends on rebuilding consumer trust in how certification institutions enforce standards. Below, we separate the recommendations that follow directly from our estimates from those that arise from interpretation and therefore need further validation.

Policymakers should pair certificate promotion with clear information on legal penalties and traceability, prioritizing these efforts on low-trust platforms and among highly aware consumers. Decoupling perception is negatively associated with trust, WTB, and WTP, which suggests that simply increasing certificate exposure is unlikely to strengthen consumer trust unless consumers also understand the institutional constraints behind the certificate. Certificate promotion should therefore be paired with clear information on legal penalties, counterfeiting costs, and government traceability. Institutional efficacy cognition is positively associated with trust and WTB but not with WTP, implying that such communication is more likely to support trust and purchase than to raise price premiums in the short term. Since quality expectation improvement shows the largest indirect coefficient, communication should highlight how the certification system safeguards product quality. The moderation results further show that these associations are stronger on low-trust platforms and among consumers with high food safety awareness, so certificate-related information should be prioritized regarding livestreaming e-commerce and community group buying and tailored to reach food-safety-attentive consumers.

Derived from our framework, future food safety governance should incorporate targeted actions such as cognitive indices, linking certificates to platform credit, and promoting voluntary disclosure, pending further empirical validation. For policymakers, a consumer cognition index could be incorporated into performance evaluation to track the gap between certificate exposure and institutional understanding, and a unified traceability platform could be built to verify production and penalty records, although both measures require pilot assessment before wider adoption. For e-commerce platforms, the certificate could be integrated into merchant credit scoring, certified merchants with high institutional efficacy cognition could receive traffic preferences, and relational rather than merely transactional contracts could be cultivated with consumers, yet these extensions require field or experimental validation. For producers, voluntary disclosure and investment in independently verifiable traceability systems align with our interpretation, although whether voluntary disclosure outperforms mandatory disclosure remains an open empirical question. For food safety incident management, firms could adopt voluntary recall strategies tailored to the violation type and consumer base, but the causal effects and boundary conditions of such strategies warrant further study.

## Figures and Tables

**Table 1:** Demographic Characteristics of the Sample.

Variable	Category	Frequency	Percentage (%)
Gender	Male	436	41.64
	Female	608	58.07
	Not clear	3	0.29
Age	18–29	323	30.85
	30–44	633	60.46
	45–59	84	8.02
	60 and above	7	0.67
Marital status	Married	799	76.31
	Unmarried	248	23.69
Monthly income (Less than 12,000 yuan is considered low-income, and more than 12,000 yuan is considered high-income)	low-income	793	75.74
	high-income	254	24.26
Education level (Less than a bachelor’s degree is considered a low level of education, and bachelor’s degree and above is considered a high level of education)	low education level	946	90.35
	high level of education	101	9.65

Note: N = 1047. Variables such as age, gender, marriage, income, and education are based on valid sample statistics.

**Table 2:** Descriptive Statistics of Key Variables.

Variable Type	Variable	Mean	SD	Min	Max
Dependent variables	Consumer trust index (CTI)	3.99	0.54	1	5
	Willingness to buy (WTB)	0.82	0.39	0	1
	Willingness to pay a premium (WTP)	2.42	0.90	1	5
Core explanatory variables	Decoupling perception	0.45	0.50	0	1
	Institutional efficacy cognition	3.06	1.21	0	5
Mediating variables	Perceived risk reduction	4.04	0.82	1	5
	Quality expectation improvement	4.14	0.66	1	5
	Trust repair	4.05	0.76	1	5
Moderating variables	Platform type	0.43	0.49	0	1
	Education	0.10	0.30	0	1
	Income	0.24	0.43	0	1
	Food safety awareness	3.99	0.57	1	5
Control variables	Online purchase frequency	2.76	0.85	1	5
	Health awareness	4.11	0.46	1	5
	Time preference	1.98	0.93	1	6

Note: N = 1047. Decoupling perception, willingness to buy, platform type, education, and income are binary variables. Willingness to pay a premium, online purchase frequency, and time preference are ordinal variables. The remaining variables are continuous.

**Table 3:** Baseline Regression Results.

Variable	CTI (OLS)	WTB (Probit)	WTB (Logit)	WTP (Ordered Probit)	WTP (Ordered Logit)
Decoupling perception	−0.0984 *** (0.0304)	−0.2157 ** (0.0977)	−0.3772 ** (0.1738)	−0.2287 *** (0.0696)	−0.3924 *** (0.1220)
Institutional efficacy cognition	0.0638 *** (0.0123)	0.1869 *** (0.0413)	0.3351 *** (0.0746)	−0.0337 (0.0282)	−0.0390 (0.0500)
Health awareness	0.4310 *** (0.0338)	0.5256 *** (0.1024)	0.9048 *** (0.1776)	0.2154 *** (0.0777)	0.4044 *** (0.1383)
Online purchase frequency	0.0801 *** (0.0184)	0.1674 *** (0.0625)	0.2964 *** (0.1125)	0.3260 *** (0.0427)	0.6012 *** (0.0764)
Time preference	−0.0176 (0.0160)	−0.0555 (0.0517)	−0.1107 (0.0910)	0.0095 (0.0365)	0.0415 (0.0638)
Gender group 2 (Female)	0.0563 ** (0.0300)	−0.0474 (0.0976)	−0.0641 (0.1733)	−0.1441 ** (0.0686)	−0.2928 *** (0.1205)
Gender group 3 (Not clear)	0.2181 (0.2747)	Omitted	Omitted	−0.1285 (0.6293)	0.0424 (1.1256)
Age group 2 (30–44)	0.0083 (0.1841)	−0.1191 (0.1345)	−0.2219 (0.2391)	0.0135 (0.0923)	0.0104 (0.1602)
Age group 3 (45–59)	0.0007 (0.1811)	−0.3101 (0.1992)	−0.5130 (0.3517)	−0.0412 (0.1466)	−0.1353 (0.2585)
Age group 4 (60 and above)	0.0306 (0.1867)	−0.3554 (0.5553)	−0.6548 (0.9155)	−0.1023 (0.4247)	−0.2035 (0.6773)
Marital status (Marriage 2)	−0.0541 (0.0442)	−0.0355 (0.1441)	−0.0584 (0.2539)	0.0334 (0.1013)	−0.0287 (0.1762)
Constant	1.9650 *** (0.2424)	−1.6430 *** (0.5057)	−2.8842 *** (0.8856)	Cut points/ thresholds	Cut points/ thresholds
Observations	1047	1044	1044	1047	1047
R^2^/Pseudo R^2^	0.2516	0.0908	0.0897	0.0428	0.0466
F/LR chi2	31.63 ***	90.44 ***	89.37 ***	112.15 ***	121.92 ***

Note: Standard errors are in parentheses. *, **, and *** denote significance at the 10%, 5%, and 1% levels, respectively. For the ordered models, cut points or thresholds are not reported. Gender group 1 (Male) and Age group 1 (18–29 years) are the reference groups and are omitted. Gender group 3 is automatically omitted in the WTB models due to perfect prediction.

**Table 4:** Average Marginal Effects from the Probit and Ordered Probit Models.

Variable	dy/dx (AME)	Std. Err.	z	*p*-Value	95% CI
Panel A: Willingness to buy (WTB, Probit model)					
Decoupling perception	−0.0520 **	0.0234	−2.22	0.027	[−0.0979, −0.0061]
Institutional efficacy cognition	0.0451 ***	0.0098	4.61	0.000	[0.0259, 0.0642]
Panel B: Highest willingness to pay a premium (WTP = 5, Ordered Probit model)					
Decoupling perception	−0.0032 **	0.0015	−2.18	0.029	[−0.0061, −0.0003]
Institutional efficacy cognition	−0.0005	0.0004	−1.10	0.271	[−0.0013, 0.0004]

Note: N = 1044 for Panel A and N = 1047 for Panel B. ** *p* < 0.05, *** *p* < 0.01. Reported figures are the average marginal effects of each variable on the probability of consumers’ behavior.

**Table 5:** Robustness Test Results of Propensity Score Matching (PSM).

Dependent Variable	ATET Coefficient	Robust Standard Error	z-Value	*p*-Value	95% Confidence Interval
Consumer trust index	−0.1027 ***	0.0366	−2.80	0.005	[−0.1745, −0.0309]
Willingness to buy	−0.0636 **	0.0325	−1.96	0.050	[−0.1272, 0.0001]
Willingness to pay a premium	−0.1045 *	0.0604	−1.73	0.084	[−0.2228, 0.0139]

Note: ATET = average treatment effect on the treated. The matching method is 1:3 nearest-neighbor matching. Robust standard errors are reported in the column “Robust Standard Error.” ***, **, and * denote significance at the 1%, 5%, and 10% levels, respectively. The 95% confidence intervals are reported in brackets.

**Table 6:** Balance Test of Covariates Before and After Propensity Score Matching.

Variable	Standardized Bias (Before Matching)	Standardized Bias (After Matching)	Variance Ratio (Before Matching)	Variance Ratio (After Matching)
Online purchase frequency	−0.433	−0.008	1.004	1.041
Health awareness	−0.321	0.025	1.422	1.021
Time preference	−0.085	0.063	0.782	1.266
Gender group 2 (Female)	−0.058	−0.020	1.019	1.006
Gender group 3 (Not clear)	−0.026	0.065	0.601	—
Age group 2 (30–44)	−0.132	−0.038	1.057	1.012
Age group 3 (45–59)	0.054	0.076	1.181	1.274
Age group 4 (60 and above)	0.129	0.102	7.122	3.567
Marital Status (Marriage 2)	0.210	−0.045	1.295	0.960

Note: “—“ indicates that the variance ratio cannot be computed due to insufficient observations in one group.

**Table 7:** Results of the Three-Step Mediation Analysis.

Variable	Total Effect c	Path a1	Path a2	Path a3	Direct Effect b1	Direct Effect b2	Direct Effect b3
Institutional efficacy cognition	0.0653 *** (0.0124)	0.0493 ** (0.0205)	0.0795 *** (0.0161)	0.0772 *** (0.0182)	0.0468 *** (0.0097)	0.0282 *** (0.0100)	0.0284 *** (0.0089)
Perceived risk reduction					0.3760 *** (0.0148)		
Quality expectation improvement						0.4667 *** (0.0190)	
Trust repair							0.4780 *** (0.0150)
Control variables	Yes	Yes	Yes	Yes	Yes	Yes	Yes
Constant	1.7381 *** (0.1478)	2.0110 *** (0.2442)	2.3155 *** (0.1922)	1.5586 *** (0.2174)	0.9820 *** (0.1197)	0.6575 *** (0.1255)	0.9931 *** (0.1078)
Observations	1047	1047	1047	1047	1047	1047	1047
R-squared	0.2440	0.0825	0.1224	0.1606	0.5356	0.5224	0.6176
F-statistic	33.44 ***	9.32 ***	14.45 ***	19.82 ***	108.50 ***	102.91 ***	151.93 ***

Note: Standard errors are in parentheses. *, **, and *** denote significance at the 10%, 5%, and 1% levels, respectively. For the ordered models, cut points or thresholds are not reported.

**Table 8:** Bootstrap Test Results of Mediation Effects (5000 Replications).

Mediation Path	Indirect Effect	Bootstrap Standard Error	95% Confidence Interval (Percentile Method)	*p*-Value
Perceived risk reduction	0.0185	0.0075	[0.0038, 0.0332]	0.013
Quality expectation improvement	0.0371	0.0076	[0.0224, 0.0522]	0.000
Trust repair	0.0369	0.0088	[0.0190, 0.0540]	0.000

Note: N = 1047; The Bootstrap is set as 5000 repeated samples, and the effective estimation times are 4764. A total of 236 bootstrap replicates failed to converge, yielding 4764 valid samples due to sparse subgroup observations (e.g., respondents aged above 45). This is common and does not affect result validity. None of the 95% confidence intervals contain 0.

**Table 9:** Heterogeneity Analysis: Moderating Effect Regression Results.

Variable	Consumer Trust Index
**Core explanatory variables**	
Decoupling perception	−0.0568 * (0.0297)
Institutional efficacy cognition	0.0576 *** (0.0125)
**Interaction terms (moderating effects)**	
IEC × Platform type	−0.0167 * (0.0092)
IEC × Education	−0.0124 (0.0152)
IEC × Income	−0.0022 (0.0110)
IEC × Food safety awareness	0.0931 *** (0.0105)
**Control variables**	
Online purchase frequency	0.0572 *** (0.0185)
Health awareness	0.2190 *** (0.0403)
Time preference	−0.0155 (0.0155)
Gender group 2 (Female)	0.0539 * (0.0290)
Gender group 3 (Not clear)	0.2317 (0.2645)
Age group 2 (30–44)	0.0155 (0.0392)
Age group 3 (45–59)	0.0372 (0.0618)
Age group 4 (60 and above)	−0.0081 (0.1773)
Marital status	−0.0194 (0.0430)
Constant	2.8480 *** (0.1850)
Model statistics	
Observations (N)	1047
F-statistic	30.76 ***
R-squared	0.3092
Adjusted R-squared	0.2991

Note: Standard errors are in parentheses. *, **, and *** denote significance at the 10%, 5%, and 1% levels, respectively. Food safety awareness is mean-centered. Platform type, Education, and Income are dummy variables (0/1).

## Data Availability

The data presented in this study are available on request from the corresponding author. The data are not publicly available due to privacy restrictions.

## Abbreviations

The following abbreviations are used in this manuscript:

CCC	Commitment Certificate of Compliance
IEC	Institutional Efficacy Cognition
PSM	Propensity Score Matching
WTP	Willingness to pay a premium
